# Repair of a Perforated Sinus Membrane with a Subepithelial Palatal Conjunctive Flap: Technique Report and Evaluation

**DOI:** 10.1155/2012/489762

**Published:** 2012-07-04

**Authors:** S. A. Gehrke, S. Taschieri, M. Del Fabbro, S. Corbella

**Affiliations:** ^1^Department of Research, Biotecnos, Rua Bozano, 571-97015-001 Santa Maria, RS, Brazil; ^2^IRCCS Istituto Ortopedico Galeazzi, Università Degli Studi di Milano, Milan, Italy

## Abstract

The maxillary sinus grafting procedure has proven to be an acceptable modality for bone augmentation to provide a base for endosseous implants, routinely used for the rehabilitation of posterior maxilla. Perforation of the membrane is the most common complication in this type of procedure. This paper presents a technique for repairing a perforated Schneiderian membrane with a conjunctive connective tissue graft harvested from the palate and shows the histological and radiographic evaluation of the results. Ten consecutives cases with the occurrence of membrane perforation were included in this study. All were repaired with a flap of tissue removed from the palatine portion near to the surgical site. The technique is demonstrated through a clinical case. The results showed successful integration of 88.8% of the implants after 12 months from prosthesis installation. Histological evaluation of the samples showed that the use of nanocrystalized hydroxyapatite showed an adequate stimulation of boné neoformation within 6 months. Radiographic evaluation revealed a small apical implant bone loss, not compromising their anchorages and proservation. Thus, it can be concluded that the use of conjunctive technique with collected palate flap for sealing the perforation of the membrane of the sinus may have predictable result.

## 1. Introduction 

The lateral window technique described in the mid 80s [1] was introduced as a method of increasing the amount of bone in atrophic posterior maxilla to allow implant placement. The lifting of the maxillary sinus floor is currently a widely used procedure for bone augmentation of the posterior maxilla in patients who underwent alveolar bone resorption and/or maxillary sinus pneumatization [2, 3], thus increasing the possibility of rehabilitative treatment of these areas with the placement of dental implants [4]. 

Schneiderian membrane perforation was the most common complication reported for lateral sinus lifting procedure [5, 6], and this can lead to loss of graft material and sometimes of the implants, as well as causing the loss of normal physiological function of the breast [7]. 

The knowledge of process for the management of the complications of implant surgery is very important in dental practice [8]. The suture of perforations of the membrane is very difficult due to its characteristics, such as consistency [9]. However, sometimes the peroration of the membrane is not detected [10]. Various methods and techniques have been described to correct this problem, such as the use of collagen membranes, fibrin glue, or bone blades removed from donor areas [11]. 

The use of tissue removed from the palatal region has been used for correction and/or grafting of periodontal defects, because it is easily accessible and has a low morbidity, with excellent biological properties [12]. 

The purpose of this study is to describe the technique of using a subepithelial palatine flap for correction of medium-size perforations during the procedure for stabilizing the maxillary sinus graft material and for preventing its displacement into the maxillary sinus. Still, we present the results of the histologic analysis of the quality of new bone within these conditions and monitoring the behavior of these areas one year after prosthesis installation.

## 2. Material and Methods

Ten cases of sinus floor elevations were included in this study conducted in Bioface Institut, Santa Maria (Brazil). Patients were treated, if they did not show any uncontrolled systemic disease and without history of maxillary sinus diseases. All patients signed an appropriate consent form for publication and monitoring of cases. After a careful planning of each case, the patients underwent maxillary sinus graft with lateral access without the simultaneous placement of implants, as indicated and planned. Before treatment, all patients were clinically and radiographically examined by panoramic radiograph and TC scans. Every two months, clinical evaluation was performed. Prophylactic oral antibiotics were used routinely for this procedure (amoxicillin 875 mg and metronidazole 400 mg) and an anti-inflammatory (Profenid 100 mg), beginning 2 h before the procedure and continued for 7 days every 12 h. 

### 2.1. Surgical Technique Report

**Figure 1:**
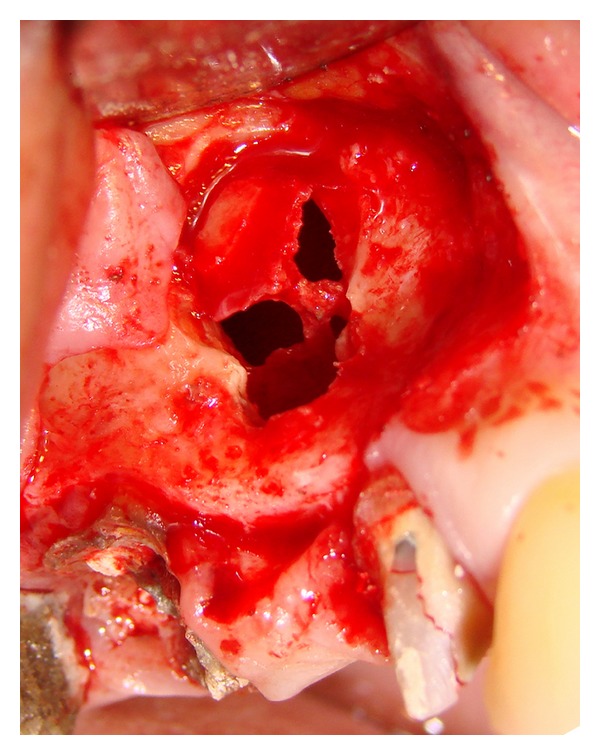
Image of the initial perforation.

All the procedures were performed under light sedation and local anesthesia. The sinus augmentation procedure was followed the technique described by Tatum et al. [13]. A horizontal antero-posterior incision was made in the alveolar crest and supplemented by buccal releasing incisions at the anterior portion of the horizontal incision. A full-thickness mucoperiosteal flap was raised and the lateral wall of the sinus was exposed. An osteotomy was made with a round bur mounted on a high-speed handpiece device with copious sterile saline irrigation. The bony wall was carefully removed through abrasion, and the elevation of the membrane began with a series of curved curettes. At some point, we observed a small or medium size (<10 mm) Schneiderian membrane perforation (Figure 1). These occurrences were not considered a reason to abort the planned augmentation procedure, but the membrane surrounding the perforation was delicately dissected with a blunt instrument, in an attempt not to increase the perforation size. 

**Figure 2:**
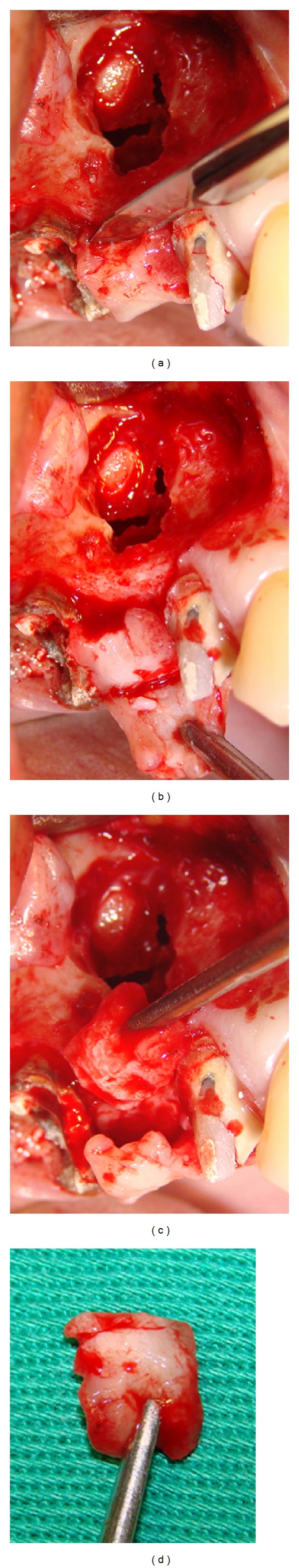
Images showing the sequence of removal of palatal tissue.

Then, a flap made only by connective tissue was removed from the palate portion, beginning the incision in the same site of the first incision of the flap prepared for the access of sinus wall, at the depth and size required to cover the perforation (Figure 2). The connective portion of the tissue was dissected from the epitelial one of the flap through the use of a 15C blade, used in an horizontal direction, parallel to the flap surface. 

**Figure 3:**
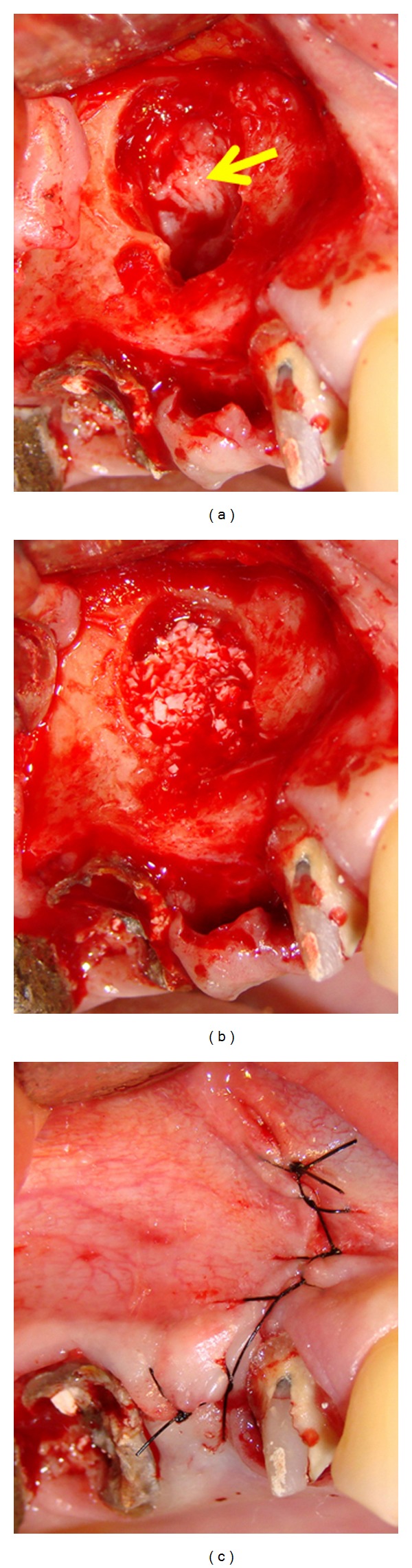
The placement of the autologous membrane, the bone graft, and the suture, respectively.

The tissue was placed and the maxillary sinus was filled by grafting material selected (Figure 3(a)). The posterior part of the cavity was grafted first, followed by the anterior portion, and finally the central area. Filling material consisted of hidroxiapatite (Nano Bone, Germany) (Figure 3(b)). This grafting protocol was used in all patients. After graft placement and compressing, the subepithelial flap was repositioned and sutured with continued sutures (Figure 3(c)).

### 2.2. Postoperative Care

Patients were advised not to blow their noses and to sneeze opening the mouth for 1 week after surgery. Patients were also instructed not to wear their dentures for 2-weeks postoperatively. Finally, sutures were removed after 7–10 days from surgery. 

After 6 months, a total of 18 tapered dental implants were placed in the prepared sites 1 mm below the bone crest. The preparation of the fixture sites was undertaken using surgical guides based on wax-up models and according to the standard clinical procedures for the implant system (Implacil DeBortoli, São Paulo, Brazil).

### 2.3. Histologic Evaluation

**Figure 4:**
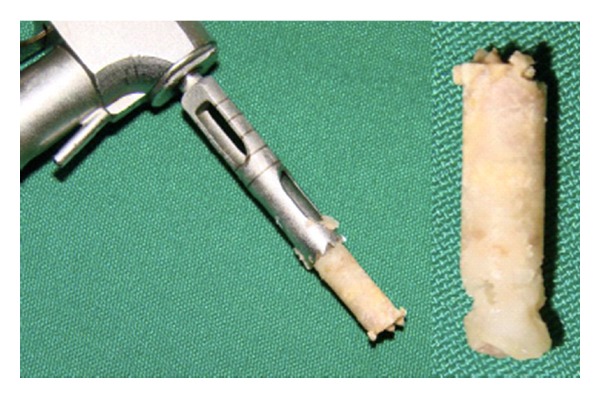
Picture of the bone fragments collected from the grafted areas for histological study.

Patients had the surgical bed initially prepared with a trephine of 2.8 mm external diameter and 2 mm internal diameter to collect the tissue sample for histological studies (Figure 4). 

The processing and the histologic measurements were performed by an experienced and calibrated, blinded examiner. Samples were fixed in 4% buffered formalin for 24 hours, dehydrated using ascending grades of alcohol (80%, 90%, 100%) and xylol, and embedded in paraffin. Sections with 2 *μ*m thickness were made for each sample. The sections were treated with xylol and a series of decreasing concentrations of alcohol (100%, 90%, 80%), immersed in distilled water, stained in hematoxylin-eosin, and observed under a light microscope (E200—Nikon, Japan) to assess morphologic aspects. The histologic characteristics of bone formation were described. 

### 2.4. Radiographical Evaluation

**Figure 5:**
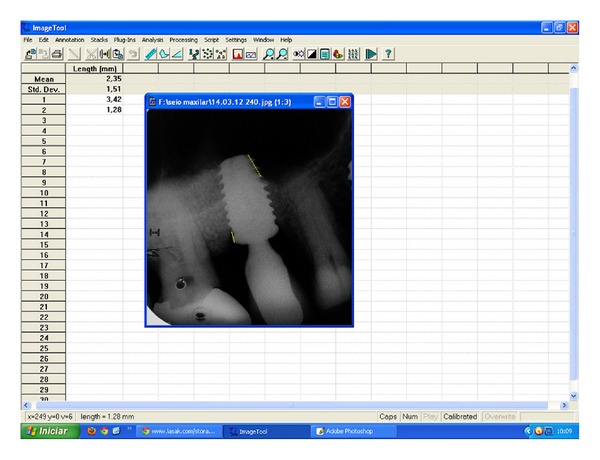
Image of the measurements being made with the review program Image Tool 3.0 for Windows.

The sites were observed radiographically after implant placement, 4 months before the beginning of the prosthetic phase and 12 months after installation of the prosthesis. Radiographs were taken using a parallel technique and the use of individualized radiograph holder. The entity of bone-to-implant contact were made with the software Image Tool 3.0 for Windows (Figure 5). These assessments was made, blindly for patients characteristics, considering the chosen radiographs by a very experienced professional (ST). No magnification devices were used for the radiographs evaluation because the used software allowed a digital zoom of the image itself.

### 2.5. Statistical Analysis

The differences between 4 and 12 months in terms of presence of bone around implants were evaluated with a Students *t*-statistic (*P* < 0.05).

## 3. Results

Sinus membrane perforations that occurred during surgical procedures were generally small with a mean diameter of 5 mm. All of them occurred during the detachment from the sinus walls.

After 6 months, two implants in one patient failed, because they were not osseointegrated and they were removed. Thus, the success rate was 88.8%. In other cases, the results showed an adequate new bone formation in patients treated with the described technique. No case had a postoperative complication in both the first and second surgical phase. 

**Figure 6:**
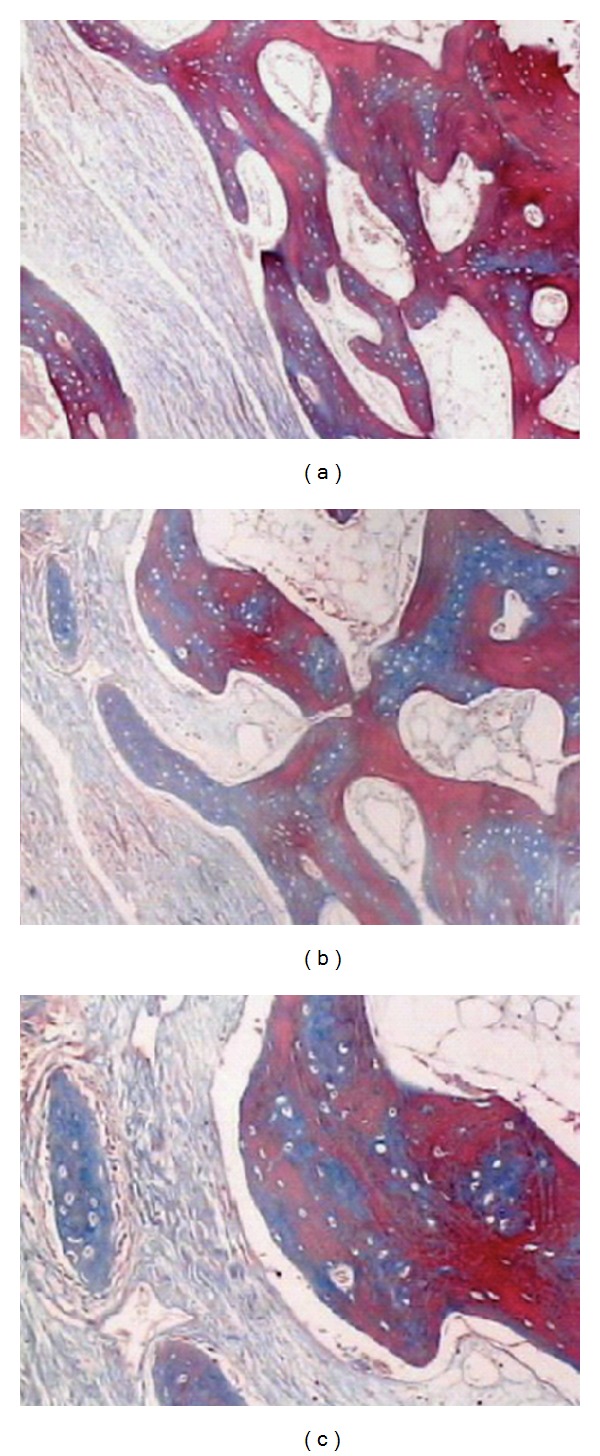
Images showing bone growth in different areas of the sample, with 40x magnification ((a)-(b)) and a 100x magnification (c). We can see the formation of fibers surrounding the “islands” of ossification. Masson's trichrome staining.

Histologically, the samples showed a new bone formation consistent with the period studied, demonstrating that the material used for grafting promoted good bone quality formation, although the amount of resorption of the material showed a very efficient integration (Figure 6). 

**Figure 7:**
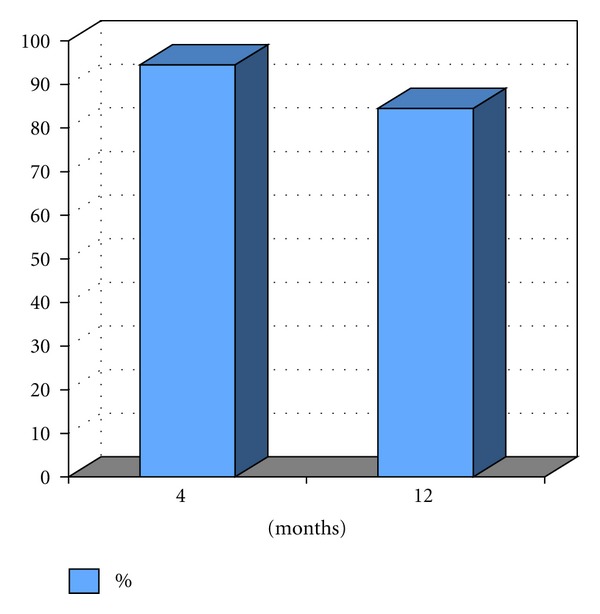
Percentage of implant portion included in bone 4 months after implantation and 12 months after prosthesis installation.

Radiographically, the measures showed a good maintenance of bone formation, as shown in the graph of Figure 5, but in most cases there is a small loss of bone more frequently in the apical portion of the implants. The presence of bone tissue around implants installed in these areas was 94.5 ± 5.3% after 4 months of implant placement and 84.5 ± 6.7% after 12 months of installation of the prosthesis on the implants, showing no a significant loss even after receiving the implant loads (*P* = 0,087) (Figure 7).

## 4. Discussion 

The present study showed that the bone graft survival in the maxillary sinus after sinus membrane perforation can be obtained after correction with a flap of tissue removed portion of the palate. 

Grafting of the maxillary sinus is a method for reaching sufficient bone height for posterior maxillary implant placement and has proven to be a highly successful method and to give predictable results [14, 15]. Sinus floor elevation procedures are routinely performed, although the function of the maxillary sinus is not clearly understood. Some of its functions might be adding resonance to the voice and some degrees of olfactory function, warming, and humidifying inspired air, as well as reducing the weight of the skull [5, 14]. 

The most commonly reported intraoperative complication of sinus augmentation is membrane perforation [15–18]. It has been reported to occur in 7–35% of sinus floor elevation procedures [14, 15, 18]. The presence of anatomic variations as well as technical factors in the region of the sinus floor can cause complications during such procedures [5, 19]. In the present study, ten cases were included where the perforation occurred during the surgical procedure. 

It may be reasonable to assume that there is a correlation between implant failure and sinus membrane perforation. In 104 cases, sinus lift surgery was complicated by perforation of the sinus membrane, which was treated using different techniques and materials intended to act as a barrier between the sinus cavity and the site of graft placement [20].

Several clinicians have recommended the use of a resorbable collagen membrane for repairing the perforated sinus membrane, and the reported implant success rate in nonperforated sites was 100%, while in perforated sites it was 69.56% [17]. Our study described an alternative for repairing of sinus membrane perforation with the use of a flap of tissue removed portion of the palate, which presented after one-year followup after prosthesis installation, an implant success rate of 88.8%. The use of an autologous connective tissue graft may be hypothesized to be more biocompatible and better tolerated by patients than other nonautologous materials. Furthermore, the autologous graft demonstrated a deep adherence to the sinus membrane tissue, and this could be useful during perforation management.

A classification for the perforated sinus membrane based on location and difficulty to repair can be described: class I perforation is a perforation that occurs at any point along the most apical wall of the prepared sinus window; class II perforations occur along the lateral or crestal aspects of the prepared sinus window and are further subdivided according to their position; class III perforations occur at any location within the body of the prepared sinus window [19, 21]. Pikos described sinus perforation by size: small (5 to 10 mm) and large (greater than 10 mm) [22]. As suggested by the results of the present study, minor membrane perforations, may not play a significant role in the clinical outcome. However, it appears that the size of the membrane perforations is related to the prognosis of the implants placed.

Previous reports suggested that larger perforations represent an absolute contraindication to the continuation of surgery [10]. Schwartz-Arad et al. [18] found no relation between membrane perforations or postoperative complications and implant survival. In our study, cases with perforations bigger than 10 mm were treated, and it was clinically observed that the grafted soft tissue promotes an easier and better stability at the site of perforation. 

It has been proposed that the regenerative result of the bone-grafting procedure is inferior following sinus membrane perforations and that simultaneous implant placement should not be performed following repairing of severe perforations [15]. According to the results of the present study, membrane perforation should not be considered an absolute contraindication for simultaneous implant placement. 

Various grafting materials have been used during sinus augmentation procedures, including autogenous bone, freeze-dried bone allografts, xenografts, hydroxyapatite, tricalcium phosphate, or a combination of these materials [15, 17, 23–26] and bone morphogenetic protein [5]. The quantity and quality of the bone graft available from the mandible seems to be sufficient and may avoid the need to harvest the bone from an extraoral site to permit sinus grafting and simultaneous implant placement [20]. In our series, a hidroxyapyatite nanocristalizated was used and has proved to be an adequate grafting material, and it was also confirmed by histological results. 

## 5. Conclusion 

The sinus membrane perforation is the most common intraoperative complication associated with the procedures for maxillary sinus elevation and grafting. Sinus membrane perforations may be adequately reconstructed and covered, and therefore they are not an absolute contraindication to the continuation of surgery, provided that they do not allow the passage of graft material inside the maxillary sinus. The use of a connective flap grafted from the palate area is a good alternative. So, the overall survival rate of implants placed under reconstructed membranes was 88,8% after 12 months. A hidroxyapatite nanocristalizated (nano bone) constitutes a viable alternative as an augmentation material for this type of procedure. The maintenance bone around the implants placed in these areas was 94.5 ± 5.33% after 4 months of implant placement and 84,5 ± 6.74% after 12 months of installation of the prosthesis on implants.

More comparative clinical trials with wider sample size and adequate randomization may be necessary to validate this technique and to evaluate the advantages and disadvantages in comparison with other surgical procedures.
